# Severe Hypercalcemia Revealing Acute Lymphoblastic Leukemia: A Case Report

**DOI:** 10.7759/cureus.100213

**Published:** 2025-12-27

**Authors:** Ibtissam Maach, Meriem M El Achiwi, Karima Ryouni, El Alaoui Mounia, Noufissa Benajiba

**Affiliations:** 1 Pediatrics, Hôpital Mère-Enfant Abderrahim Harouchi, Casablanca, MAR; 2 Pediatrics, Faculty of Medicine and Pharmacy, Hassan II University of Casablanca, Casablanca, MAR; 3 Pediatrics, Abderrahim Harouchi Mother and Child Hospital, Ibn Rochd University Hospital Center, Casablanca, MAR

**Keywords:** acute hypercalcemia, acute lymphoblastic leukemia (all), blood cancer, malignant hypercalcemia, pediatric leukemia

## Abstract

Hypercalcemia is a rare metabolic disorder in children, with a wide range of potential etiologies, some of which can be life-threatening. We report the case of a 2.5-year-old male child admitted for progressive lower limb muscle weakness that began one month prior to admission, associated with abdominal pain and vomiting. Initial investigations revealed severe hypercalcemia at 21.2 mg/dL (5.28 mmol/L), accompanied by hypercalciuria, hypophosphatemia, and vitamin D deficiency. The patient was transferred to pediatric intensive care due to the onset of malignant hypercalcemia, altered consciousness, and ECG-documented arrhythmia. Treatment included aggressive intravenous hydration and administration of bisphosphonates, which partially corrected the calcium imbalance. An extensive etiological workup was conducted and revealed no endocrine, renal, or infectious abnormalities. A complete blood count showed microcytic hypochromic anemia without additional hematological anomalies, and the initial bone marrow aspirate was normal. Radiography of the forearm revealed demineralization of the distal metaphyses, while imaging of the tibia identified metaphyseal osteolytic lesions in the distal femur, proximal tibia, and fibula, with localized cortical lysis. Although the initial bone marrow aspirate was normal, the combination of osteolytic lesions and progressive skeletal demineralization was highly atypical for benign conditions and increasingly suggestive of an underlying malignant process. These imaging findings prompted repeat bone marrow evaluation, which ultimately confirmed B-cell acute lymphoblastic leukemia. Pancytopenia developed after the third bone marrow examination, which had confirmed the diagnosis, reflecting a rapid shift from the initially normal hematologic status. Chemotherapy was initiated promptly, and the patient responded favorably, with complete remission documented on follow-up marrow evaluation. This case underscores that hypercalcemia, whether isolated or accompanied by osteolytic lesions, may constitute the sole initial clinical manifestation of acute lymphoblastic leukemia (ALL) in children. The absence of circulating blasts, together with a completely normal initial bone marrow aspirate, demonstrates how ALL can present deceptively in its early stages. Recognizing that early marrow evaluations may be falsely reassuring is crucial in persistent or atypical presentations, highlighting the importance of repeating bone marrow assessment when clinical or imaging findings raise concern.

## Introduction

Hypercalcemia is a rare but potentially serious metabolic abnormality in pediatric patients [1]. Its etiologies are diverse, encompassing endocrine, renal, infectious, drug-induced, and neoplastic origins. Although acute lymphoblastic leukemia (ALL) is the most common type of leukemia in children, its presentation with initial hypercalcemia is exceptionally rare, with an estimated prevalence of 0.4-1.3% among children diagnosed with ALL [1]. Hypercalcemia in ALL has been particularly associated with certain cytogenetic subtypes, which carry a poor prognosis [2]. The clinical presentation of hypercalcemia is varied and often non-specific, including symptoms such as vomiting, abdominal pain, and muscular weakness. However, it can progress to more severe complications such as seizures, cardiac arrhythmia, or renal failure, sometimes requiring intensive care unit management [3]. In hematologic malignancies, hypercalcemia arises through two principal mechanisms: a humoral mechanism mediated by parathyroid hormone-related peptide (PTHrP), typically characterized by suppressed PTH and 1,25(OH)₂D levels; and osteolytic hypercalcemia, resulting from direct bone infiltration and local release of osteoclast-activating cytokines [2]. Importantly, some pediatric ALL cases may initially lack circulating blasts or even present with a normal early bone marrow aspirate, potentially delaying diagnosis. Our case illustrates this challenge: a 2.5-year-old child with severe hypercalcemia and osteolytic lesions despite an initially normal marrow, ultimately revealing a diagnosis of acute lymphoblastic leukemia. This atypical presentation underscores a rare and clinically instructive presentation within pediatric ALL.

## Case presentation

We report the case of a 2.5-year-old boy admitted for progressive, proximal, lower limb muscle weakness that had been evolving over a one-month period, accompanied by diffuse abdominal pain and intermittent vomiting. Upon initial examination, the child was conscious (Glasgow Coma Scale score: 15/15) and hemodynamically and respiratory stable. A complete physical examination was performed, including neurological, cardiopulmonary, and abdominal assessment, which revealed no focal deficits or signs of dehydration, and no hepatomegaly nor splenomegaly. Functional impairment of the left lower limb was observed, attributable to pain-limited joint mobilization.

Initial laboratory investigations are summarized in Table 1. They revealed severe hypercalcemia with associated hypercalciuria, hypophosphatemia, and vitamin D deficiency, in addition to hypochromic microcytic anemia and a white blood cell count that was marginally elevated relative to age-specific norms, without clear clinical significance.

**Table 1:** Initial laboratory investigations

Parameter	Result	Reference range	Unit
Calcium (serum)	21.2	9.0 – 10.5	mg/dL
Calcium (serum, SI conversion)	5.3	2.2 – 2.6	mmol/L
Phosphate (serum)	19	35 – 45	mg/L
Vitamin D (25-hydroxyvitamin D)	23	30 – 100	ng/mL
Urinary calcium (calciuria)	263	< 200	IU/L
Red blood cell count	3.4	4.0 – 5.2	× 10⁶/µL
Hemoglobin	7.2	12 – 16	g/dL
Hematocrit	25.4	36 – 46	%
Mean corpuscular volume (MCV)	66.8	80 – 96	fL
Mean corpuscular hemoglobin (MCH)	21.6	27 – 33	pg
Mean corpuscular hemoglobin concentration (MCHC)	30.5	32 – 36	g/dL
White blood cell count	12.09	4.0 – 10.0	× 10³/µL
Neutrophils	5.39	1.8 – 7.5	× 10³/µL
Lymphocytes	5.97	1.0 – 4.0	× 10³/µL
Monocytes	0.85	0.2 – 0.8	× 10³/µL
Platelets	224	150 – 400	× 10³/µL

**Figure 1:**
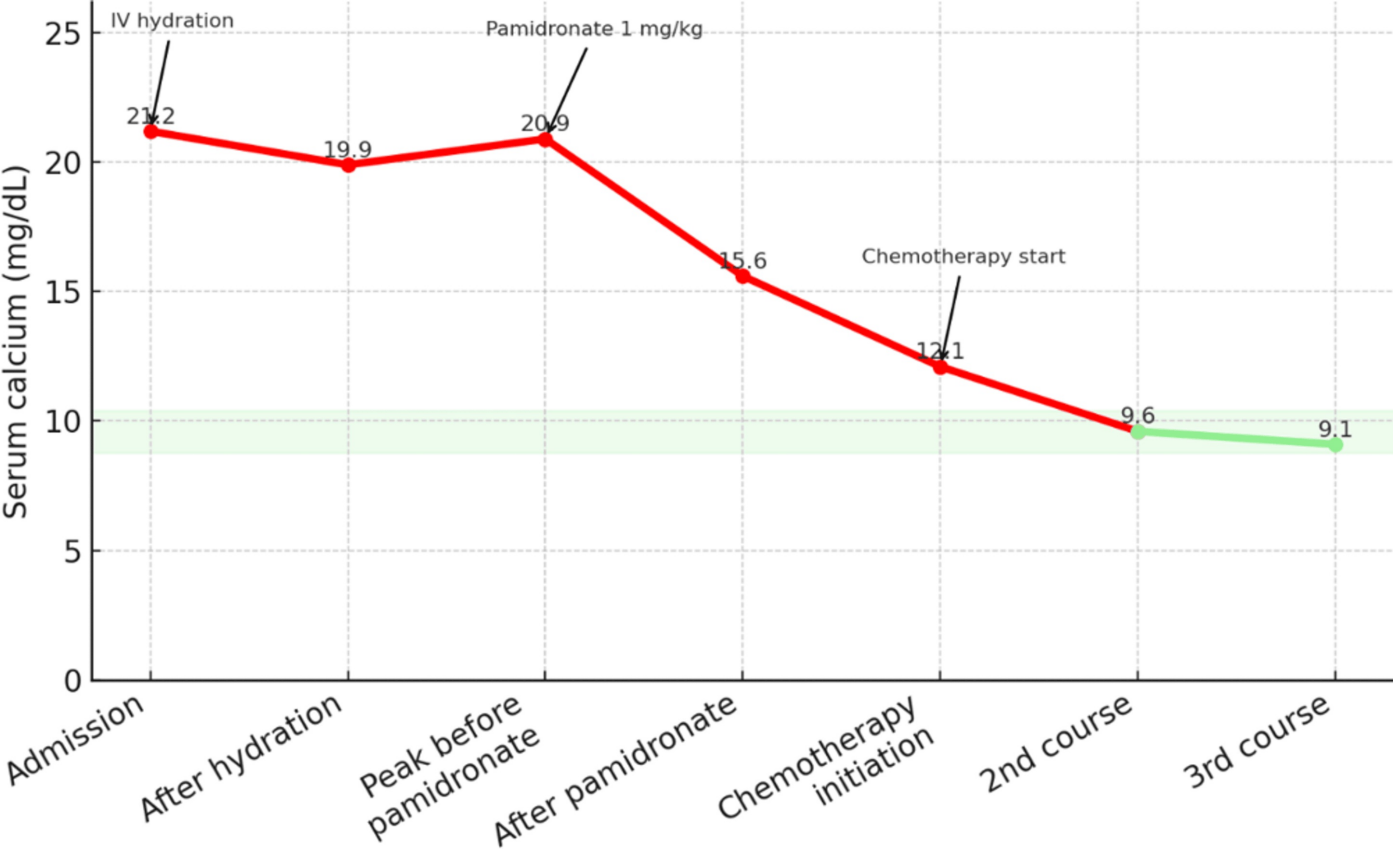
Serum calcium trend over time with key therapeutic interventions. Red line segments indicate hypercalcemic values above the normal range. Light green line segments represent values within the normal range. The shaded green band represents the normal calcium range (8.8–10.4 mg/dL). Arrows mark the timing of major therapeutic interventions: intravenous hydration, pamidronate administration, and initiation of chemotherapy.

Within 72 hours of admission, the patient developed neurological deterioration with altered consciousness, coinciding with peak calcium levels. Electrocardiographic monitoring revealed sinus bradycardia associated with a markedly shortened QT interval, prompting transfer to the pediatric intensive care unit. Intensive intravenous rehydration and bisphosphonate therapy were initiated (pamidronate at a dose of 1 mg/kg), leading to gradual biochemical improvement, although full normalization of calcium levels was not achieved (Figure 1).

To further explore the etiology, an endocrine, renal, infectious, and hematologic workup was performed (Table 2). This workup excluded common secondary causes of hypercalcemia: thyroid function was normal, renal function and renal-bladder ultrasound were unremarkable, inflammatory markers showed no evidence of infection, and the initial bone marrow aspirate was normal. The patient’s medical history was otherwise unremarkable, with no history of drug or toxic exposure.

**Table 2:** Initial etiological workup results

Parameter	Result	Reference range	Unit
Thyroid-stimulating hormone (TSH)	2.35	0.5 – 4.5	mIU/L
Free thyroxine (Free T4)	1.4	0.8 – 1.8	ng/dL
Serum creatinine	5.3	3.0 – 7.0	mg/L
Blood urea	0.51	0.15 – 0.45	g/L
Estimated glomerular filtration rate (eGFR)	90	> 90	mL/min/1.73 m²
C-reactive protein (CRP)	7.1	< 10	mg/L
Bone marrow aspirate	Cellular; Normal megakaryocytes; all lineages present; no exess blasts or atypia		

Radiographic imaging with plain X-rays showed demineralization of the distal metaphyses of the forearm bones, and multiple metaphyseal osteolytic lesions in the distal femur and proximal tibia and fibula, with focal cortical lysis. 

Due to the persistence of anemia and hypercalcemia and the onset of clinical deterioration, a second bone marrow aspirate was performed one month after admission, revealing 5.75% blasts. Given the discrepancy between the progressive clinical deterioration, the radiologic abnormalities, and the inconclusive early marrow results, a third bone marrow aspirate with immunophenotyping was carried out one month later (i.e., two months after admission). This evaluation revealed 56.7% blasts, and cytomorphology and immunophenotypic markers confirmed a diagnosis of B-cell acute lymphoblastic leukemia (Table 3)

**Table 3:** Evolution of bone marrow blast percentage in relation to clinical evolution.

Time point	Clinical context	Bone marrow blasts
Admission	Before any treatment	No blasts
After 1 month	Persistent hypercalcemia and anemia, after hydration + bisphosphonates	5.75% blasts
After 2 months	Before chemotherapy initiation	56.7% blasts

Cytogenetic analysis performed on the third bone marrow aspirate showed a normal karyotype. Corticosteroid sensitivity was assessed after seven days of prednisone, according to the MARALL 06 protocol criteria (Khattab M, Zafad S, Hachim J, Benchekroun B, Mahmal I. Protocole de traitement des leucémies aiguës lymphoblastiques (MARALL 2006). Société Marocaine d’Hématologie et d’Oncologie Pédiatrique; 2006), and the patient was classified as steroid-sensitive (blast count <1000/µL). Considering the patient’s age, absence of initial neurological involvement, normal leukocyte count at diagnosis, normal cytogenetics, and documented steroid sensitivity, the case met the criteria for standard-risk classification under the MARALL 06 protocol. Induction chemotherapy included prednisone, vincristine, L-asparaginase, and daunorubicin. Pancytopenia developed shortly after the diagnostic marrow (Table 4), coinciding with progressive marrow infiltration, prior to initiating chemotherapy. Serum calcium normalized after the initial cycles of chemotherapy (Figure 1), and complete remission was confirmed by follow-up bone marrow examination.

**Table 4:** Complete blood count at the occurrence of pancytopenia, with normal ranges for a 2.5-year-old child

Parameter	Value	Units	Normal range (2.5 years)
Hemoglobin (Hb)	5.1	g/dL	11.0 – 13.5
Mean Corpuscular Volume (MCV / VGM)	92.4	fL	70 – 86
Mean Corpuscular Hemoglobin Concentration (MCHC / CCMH)	32.3	g/dL	32 – 36
Mean Corpuscular Hemoglobin (MCH / TCMH)	29.8	pg	24 – 30
White Blood Cells (WBC / GB)	3,100	/mm³	6,000 – 17,500
Neutrophils (PNN)	1,200	/mm³	1,500 – 8,500
Lymphocytes	1,460	/mm³	3,000 – 9,500
Monocytes	300	/mm³	200 – 900
Basophils	100	/mm³	< 150
Eosinophils	40	/mm³	< 500
Platelets	61,000	/mm³	150,000 – 450,000

## Discussion

Severe hypercalcemia in children is uncommon and often signals an underlying systemic disease, prompting evaluation for endocrine, inflammatory, iatrogenic, malignant or genetic causes. It is potentially life-threatening, with an estimated prevalence ranging from 0.4% to 1.2% among hospitalized children [4]. In the present case, severe hypercalcemia served as the initial manifestation of acute lymphoblastic leukemia (ALL).

Although ALL is the most frequent leukemia in children, it rarely presents with isolated hypercalcemia as an inaugural symptom. Indeed, less than 1% of pediatric ALL cases manifest hypercalcemia at diagnosis [4]. A retrospective study covering over 6,000 pediatric cancer patients at St. Jude Children’s Research Hospital over 29 years identified only 10 out of 2,816 children with ALL who had hypercalcemia, with only seven of them presenting with hypercalcemia at diagnosis [5]. In that study, hypercalcemia was defined as a serum calcium level ≥11.5 mg/dL, which helps contextualize the severity of hypercalcemia observed in our patient.

Malignant hypercalcemia in children arises predominantly through two pathophysiological mechanisms. The first involves extensive osteolytic activity driven by leukemic infiltration of bone, which promotes the local release of osteoclast-activating cytokines and leads to accelerated bone resorption. The second mechanism is humoral, in which tumor cells secrete factors, most notably parathyroid hormone-related peptide (PTHrP), that increase osteoclastic activity and renal calcium reabsorption independently of parathyroid hormone (PTH) [2,6]. In our patient, the presence of multiple metaphyseal osteolytic lesions strongly suggests a primarily osteolytic mechanism, although the absence of PTH and PTHrP measurements prevents definitive exclusion of a concomitant humoral component.

The clinical manifestations of hypercalcemia are varied and nonspecific, affecting multiple systems. Neurological signs may include lethargy, irritability, hypotonia, and- in severe cases- altered mental status or coma. Gastrointestinal symptoms include anorexia, vomiting, constipation, and abdominal pain, often accompanied by dehydration secondary to polyuria. Renal complications include polydipsia and nephrocalcinosis, with potential progression to acute renal failure if the hypercalcemia is not promptly corrected. Cardiovascular signs, though less frequent, may include bradycardia, hypertension, and ECG conduction abnormalities [7-8].

A study by Pécheux et al. highlighted the high prevalence of bone involvement-particularly osteolytic lesions-in children with ALL. These lesions may be inaugural and are often accompanied by diffuse bone pain, pathological fractures, or focal lytic lesions. They are frequently misinterpreted as rheumatologic or infectious diseases, potentially delaying diagnosis [9].

In our case, the presence of bone lesions and clinical deterioration prompted a bone marrow examination that confirmed B-cell phenotype ALL. A notable and unusual feature of this case is that the initial bone marrow aspirate was completely normal, with blasts only emerging on subsequent samples. Studies have reported that a minority of ALL cases may present with initially nondiagnostic marrow, particularly in the context of hypercalcemia or bone-dominant disease, underscoring the importance of repeated sampling when clinical suspicion persists. In our patient, pancytopenia developed only after the third marrow examination, and therefore did not contribute to the diagnostic process. This delayed hematologic involvement further highlights the deceptive early presentation of atypical ALL and the risk of diagnostic delay when relying solely on initial marrow morphology. [10].

When compared with previously reported pediatric ALL cases with hypercalcemia, our patient’s presentation shares some common features-such as young age, severe PTH-independent hypercalcemia, and the presence of multiple osteolytic lesions-yet it also displays several uncommon aspects. Osteolytic lesions are frequently reported, but the combination of extensive metaphyseal involvement with a completely normal initial bone marrow aspirate is unusual. In most series, marrow infiltration is already evident at diagnosis; delayed marrow positivity, as observed here, has been described only in a minority of cases.

In our patient, pancytopenia appeared only after the third bone marrow examination-corresponding to overt leukemic infiltration, thus represented a late marker of disease progression rather than a contributor to the diagnostic process.

Initial therapeutic management focuses on correcting hypercalcemia: intravenous hydration, loop diuretics, and, when resistant, bisphosphonate administration or dialysis in severe cases. Bisphosphonates are highly effective and generally well tolerated in children, although caution is advised due to potential interference with bone remodeling [11]. As illustrated in our case presentation, our patient showed only partial biochemical improvement after hydration and pamidronate, with serum calcium declining substantially but remaining above the normal range; complete normalization occurred only after the initiation of induction chemotherapy, underscoring that definitive correction of malignant hypercalcemia in ALL typically depends on controlling the underlying leukemia. Ultimately, the definitive treatment lies in the management of ALL itself, using established national or international protocols, and the prognosis is generally favorable in cases with prompt response to induction chemotherapy. A study by Park et al. (2016) suggests that the presence of hypercalcemia in patients with leukemia does not negatively impact prognosis [12].

## Conclusions

Hypercalcemia in children is a rare but serious metabolic emergency, with diverse and sometimes elusive underlying causes. When associated with osteolytic lesions or persistent biological abnormalities, a malignant hematologic etiology-such as ALL-should always be considered. The absence of circulating blasts or hematologic signs at initial presentation should not preclude this diagnosis. A thorough investigation, including bone marrow aspiration and immunophenotyping, is essential for timely and appropriate management. Importantly, this case underscores that an initial bone marrow aspirate may be entirely normal, with blasts appearing only on subsequent samples, which can significantly delay recognition of ALL in hypercalcemic presentations. Continuous clinical vigilance is crucial to avoid diagnostic delays and to improve the prognosis in these atypical presentations.
